# S100P Interacts with p53 while Pentamidine Inhibits This Interaction

**DOI:** 10.3390/biom11050634

**Published:** 2021-04-24

**Authors:** Revansiddha H. Katte, Deepu Dowarha, Ruey-Hwang Chou, Chin Yu

**Affiliations:** 1 Department of Chemistry, National Tsing Hua University, Hsinchu 30013, Taiwan; revankatte@gmail.com (R.H.K.); deepu.dowarha@outlook.com (D.D.); 2 Graduate Institute of Biomedical Sciences and Center for Molecular Medicine, China Medical University, Taichung 40402, Taiwan; rhchou@gmail.com; 3 Department of Biotechnology, Asia University, Taichung 41354, Taiwan

**Keywords:** S100P, p53-TAD (73 amino acids), protein-protein interactions, ^1^H-^5^N HSQC spectrum, biomolecular docking, HADDOCK program

## Abstract

S100P, a small calcium-binding protein, associates with the p53 protein with micromolar affinity. It has been hypothesized that the oncogenic function of S100P may involve binding-induced inactivation of p53. We used ^1^H-^15^N HSQC experiments and molecular modeling to study the molecular interactions between S100P and p53 in the presence and absence of pentamidine. Our experimental analysis indicates that the S100P-53 complex formation is successfully disrupted by pentamidine, since S100P shares the same binding site for p53 and pentamidine. In addition, we showed that pentamidine treatment of ZR-75-1 breast cancer cells resulted in reduced proliferation and increased p53 and p21 protein levels, indicating that pentamidine is an effective antagonist that interferes with the S100P-p53 interaction, leading to re-activation of the p53-21 pathway and inhibition of cancer cell proliferation. Collectively, our findings suggest that blocking the association between S100P and p53 by pentamidine will prevent cancer progression and, therefore, provide a new avenue for cancer therapy by targeting the S100P-p53 interaction.

## 1. Introduction

The transcription activator protein p53 plays a crucial role in regulating tumor formation, suppression, and cancer prevention. Under cellular stress conditions, the p53 protein is activated and initiates transcription of various protein whose protein products are involved in DNA repair, cell cycle arrest, or programmed cell death depending upon conditions in the cell [1,2,3]. The transcriptional ability of the p53 gene is critically monitored by a regulatory feedback loop involving the mouse double minute 2 homolog (MDM2) protein, a negative regulator of the p53 protein [4,5,6]. In addition, it is also controlled by a variety of post-translational modifications and interactions with a wide range of target signaling proteins [4]. The p53 function is demonstrated in its tetrameric form with 393 amino acid residues in each of its four identical chains. The N-terminal region, representing the transactivation domain (TAD) and comprising the first 73 residues (p53^1-73^), is intrinsically disordered [7], which facilitates conformational adaptability upon binding to multiple target proteins and confers high specificity as seen in proteins involved in signaling networks [8,9]. This conformational adaptability feature is due to the presence of about 20 amino-acid residues that undergo a transition from a disordered to ordered phase as a result of interactions with the target molecule [10,11]. The proposed interaction cause changes in the nascent helical region to yield a rigid and folded native form [12,13]. Although p53-TAD is projected as an intrinsically disordered protein, it does have local secondary motifs consisting of an alpha-helix region between the T18 and L26 amino acid residues [named p53^18-26^] and two nascent turns, termed turn I (M40 to M44) and turn II (D48 to W53) [14,15]. As the cell detects stress signals, p53 gene activation is initiated, which as a whole, is a very intricate process and a result of phosphorylation of various serine and threonine residues at its N-terminus via the action of many different protein kinases [16]. These alterations shift the respective binding affinity toward diverse target proteins that compete for the p53 association. This p53^1-73^ region associates with the MDM2 N-terminal part and thus has been extensively examined for its significant role by several techniques to elucidate its underlying mechanism of action. The p53^1-73^ region was found to have no fixed three-dimensional (3D) conformation but binding with its target partner induces a 3D conformation—which is specific to the binding partner under consideration [17]. The p53^1-73^ regions are prone to enzyme digestion even in the absence of their target protein/binding partner and are thus in a loosely folded configuration [18]. As the p53^1-73^ region is involved in binding to multiple partners, it is subjected to enzymatic processing for various post-translational modifications (PTMs) [19]. PTMs result in enhancement or inhibition of p53 interactions with its target partners thereby regulating p53 function [20,21]. The interaction with the p53 is a common feature of many S100 proteins (S100B, S100A1, S100A2, S100A6, S100A11, S100A4) [22,23]. The binding of S100 proteins to the p53 not only involves interaction with the intrinsically disordered N-terminal transactivation domain of p53 (TAD, residues 1-74) [24], but also with and an amphipathic α -helical region (residues 325-355) and adjacent intrinsically disordered domain (residues 367-388) located within the C-terminus presumably through “fly casting” [22,25,26]. Earlier, the S100P-p53 relationship was studied using pull-down experiments with the GST-wtS100P protein and data show that the S100P protein has the capability of binding both p53 and MDM2, can affect p53 phosphorylation, and alter the p53-mediated target expression [27,28]. Moreover, the S100P protein was found to interact with both, the p53 N-terminal TAD region as well as the p53 C-terminal domain [27].

The S100P protein is a member of a large family of S100 calcium-binding proteins that mediate Ca^2+^ dependent signal transduction pathways [29,30]. It has 95 amino acid residues. Becker et al. isolated and characterized S100P protein from placenta in 1992. It was assigned the designation “P” to show that it was purified first from placenta [31]. Upon binding of calcium ions S100P undergoes a conformational change that results in an exposure of a hydrophobic surface which allows the interaction with specific target proteins. The up-regulation of S100P is an early event in the development of pancreatic cancer and its expression increases throughout the progression of pancreatic intraepithelial neoplasia (PanINs) to invasive pancreatic ductal adenocarcinoma. Recent findings suggest that S100P protein expression is upregulated in a range of cancers, consisting of the colon [32], pancreatic [33], breast [34], and lung carcinomas [35]. For instance, in cancers associated with the colon and pancreas, S100P induced cancer invasion, migration, and proliferation through the stimulated receptor for advanced glycation end-products (RAGE) receptor [29,30]. In addition, the association of S100P with the Siah 1 interacting protein (CacyBP/SIP) and calcyclin-binding protein resulted in β-catenin degradation, which is responsible for tumorigenesis in various cancer types [36].

Earlier studies suggest that the numerous small molecules, such as Cromolyn, Amlexanox, tranilast, and pentamidine, bind to the S100 family proteins [37,38,39]. Pentamidine is an aromatic diamidine pharmacologically active drug approved by the FDA for protozoal diseases, including the diagnosis and prophylaxis of pneumocystis pneumonia and the treatment of trypanosomiasis and visceral leishmania in patients with AIDS [40]. Pentamidine has recently been identified as a potential anti-cancer drug, particularly in the context of melanoma, which is believed to inhibit interaction with S100B-p53 [39,41]. It was therefore of interest to study a molecular level interaction between pentamidine and S100P. Thus, in this study, we elucidated the S100P interactions with the p53^1-73^ and pentamidine and then checked the feasibility of pentamidine as an antagonist toward the S100P and p53^1-73^ interactions.

We used both ^1^H-^15^N heteronuclear single quantum coherence spectroscopy/nuclear magnetic resonance (HSQC/NMR) and molecular docking to study the molecular interactions between the S100P and p53^1-73^ regions, together with S100P and pentamidine. Our analysis indicated that the complex formation of S100P with the p53^1-73^ is successfully disrupted by pentamidine as S100P shares the same binding site for p53 and pentamidine. In addition, to prove the concept in cancer cells, ZR-75-1 breast cancer cell line, which contains wild-type p53 gene and expresses a high level of S100P, was used to determine the effects of pentamidine on cell proliferation by WST-1 assay and on the amounts of p53 protein by Western blotting. The results further provide the evidence of pentamidine efficacy in vitro by reducing cell proliferation and increasing the p53 protein level, suggesting the role of pentamidine as an effective antagonist to interfere the S100P-p53 interaction and reactivate normal p53 function. Collectively, our findings suggest that blocking the association between the S100P and p53^1-73^ regions will prevent the progression of cancer with wild type p53 and therefore provides a new avenue for cancer therapy targeting S100P-p53 interaction.

## 2. Materials and Methods

### 2.1. Protein Expression and Purification

The pET-20b vector containing cDNA for the human S100P protein was inserted into the *Escherichia coli* BL21 (DE3) strain for over-expression of the S100P protein. M9 medium was used for the production of ^15^N-labeled proteins. A previously published experimental procedure for purification was used [42]. Following purification, and prior to HSQC/NMR titrations, the pure ^15^N-labeled S100P protein fractions were buffer-exchanged with the NMR buffer consisting of 20 mM Tris-HCl, 4 mM CaCl_2,_ 100 mM KCl, and pH 7.0.

The pET-28a vector consisting of cDNA for human p53^1-73^ region protein was inserted into *E. coli* BL21 (DE3) strain for over-expression of the p53 protein. M9 medium was used for the production of the ^15^N-labeled protein. Following purification, prior to HSQC/NMR titrations, the pure ^15^N-labeled p53^1-73^ region protein fractions were buffer-exchanged with the NMR buffer (pH 7.2) containing 25 mM Tris, 114 mM NaCl, 5 mM CaCl_2_, 0.05 mM ethylenediaminetetraacetic acid (EDTA), and 1 mM Dithiothreitol (DTT).

The purity (≥95%) of the S100P and p53 proteins was verified with polyacrylamide gel electrophoresis (PAGE), and identity was confirmed with high-resolution electrospray ionization mass spectrometry (HR/ESI/MS) method.

### 2.2. NMR Titration Experiments

All of the HSQC-NMR data were recorded using a cryogenic probe fitted with a Varian 700 MHz spectrometer (Agilent Technologies, Santa Clara, California, USA) operated at 25 °C. The NMR samples were prepared in 20 mM Tris-HCl (pH 7.0), 4 mM CaCl_2_, 100 mM KCl, and 10% (*v*/*v*) D_2_O. The ^1^H-^15^N heteronuclear single-quantum correlation (HSQC) profile of 0.3 mM labeled S100P was obtained alone and in combination with its unlabeled specific partner with variable concentrations (supporting information Figures S1 and S2). The first partner was the p53^1-73^, and the second partner was the pentamidine drug molecule. Similarly, the HSQC/NMR profile of the 0.3 mM labeled p53^1-73^ was obtained alone and in combination with variable concentrations of its unlabeled partner S100P (supporting information Figure S3). The cross-peak assignment data for S100P and p53^1-73^ were acquired from the Biological Magnetic Resonance Databank (BMRB) and corresponded to 17866 and 17760. Equation 1 was used to calculate the weighted differential centrifugal sedimentation (DCS) values for backbone ^1^H and ^15^N resonance measurements [43]. The SPARKY program was used to process and analyze the resulting NMR data [44].

Chemical shift difference:

(Δδ) = [(δ^1^HN)^2^ + 0.2 (δ^15^N)^2^]^1/2^ (1)

The dissociation constants (K_d_) for the S100P-p53^1-73^ and the S100P-pentamidine complexes were calculated by using the chemical shift perturbation data. Equation (2) [45] was used to determine the above-mentioned K_d_ value.

(2) $$\Delta \delta_{obs}=\Delta \delta_{\max}\{{\left([P{]}_{t}+\left[L{]}_{t}+K_{d}\right)-[{\left({\left[P\right]}_{t}+{\left[L{]}_{t}+K_{d}\right)}^{2}-4[P{]}_{t}[L\right]}_{t}\right]}^{\frac{1}{2}}\}÷2{\left[P\right]}_{t}$$

in which [L]_t_ is the total concentration of ligand (p53-TAD) and [P]_t_ is the total concentration of protein (S100P), Δδ_obs_ represents the change observed in the shift amid the free and bound-form, and Δδ_max_ indicates the maximum chemical shift change detected for the specific peak under consideration.

### 2.3. Modeling

For the prediction of protein complex formation, the computer-assisted data driven HADDOCK 2.2 program was utilized by using HSQC/NMR data as restraints [46,47,48]. For HADDOCK-assisted calculations of the complex structure of the S100P-p53^1-73^ and S100P-pentamidine, cross-peaks as input parameters were selected based on a decrease in cross-peak intensity and significant changes in cross-peak positions. Residues were assigned as active and passive based on the NACCESS program output. Active residues had ≥40 percent accessible areas, and the remaining residues were considered passive. The residues of S100P consists of T6, G9, V14, S16, M37, G43, Q46, D50, D52, A53, E73, F74, I75, V76, F77, A79, A80, S83, A84, K91, A92, and G93 while p53-TAD residues consist of T18, F19, S20, D21, W23, K24, L25, L26, L28, and N29. The starting PDB file for S100P was obtained from PDB code: 1J55, and for p53^17-29^ was derived from the PDB code: 1YCR. The rigid-body energy minimization stage resulted in a 2000 structure, and additional 2000/200/200 models corresponding to the it0/it1/water refinement stages were created. At the end of this process, the best 200 models were clustered as per their interface-ligand root-mean-square deviation (RMSD) values with a cut-off of 5 Å, and all the clusters were given a HADDOCK score. PyMOL software (version 2.3.3) was used to visualize the created complexes through the HADDOCK program [49].

Similarly, the complex model of the S100P-pentamidine was created employing the HADDOCK program. The residues from the S100P comprised G9, I12, D13, V14, G43, Q46, G48, I75, A79, S83, and A84. The PDB coordinates were acquired from the PDB code: 1J55 for S100P and the pentamidine drug coordinates were taken from the Drug Bank, accession number DB00738.

### 2.4. Assay of Cell Proliferation

Cell proliferation was determined by WST-1 assay (Roche Applied Science). WST-1 (4-[3-(4-iodophenyl)-2-(4-nitrophenyl)-2H-5-tetrazolio]-1,3-benzene disulfonate) produces a water-soluble formazan dye upon reduction by mitochondrial dehydrogenase enzymes in viable cells. The released soluble formazan in the medium is correlated to the number of viable cells. The more cells, the more dehydrogenase enzymes available to reduce WST-1 to water-soluble formazan. To perform the WST-1 assay, ZR-75-1 human breast cancer cells were seeded at a concentration of 5 × 103 cells/well on a 96-well plate in a day before experiments. Subsequently, the cultured cells were treated with or without increasing concentrations from 5 to 100 μM of pentamidine for 48 h. Afterwards, 1/10 volume of WST-1 reagent was added to the wells and incubated at 37 °C for another 4 h before harvesting. The plate was agitated by gentle shaking to mix the medium in the wells. The absorbance of the released soluble formazan in the wells were measured at 450 nm by the Synergy 2 microplate reader (Bio-Tek Instruments, Inc.). The relative cell numbers were determined by the relative absorbance comparing to that from control treatment. The statistical analysis was performed by a *t*-test and the *p* value < 0.05 was considered statistically significant.

### 2.5. Western Blotting

Cells were washed twice with phosphate buffer saline (PBS, containing 137 mM NaCl, 2.7 mM KCl, 10 mM Na_2_HPO_4_, 2 mM KH_2_PO_4_). Cell lysate was extracted by sonication in RIPA Buffer (50 mM Tris at pH 7.5, 150 mM NaCl, 1 mM EDTA, 0.25% Na-deoycholate, 1% NP-40, 1 mM NaF, 1 mM Na_3_VO_4_, 1 mM PMSF, 1 μg/mL aprotinin). The soluble protein extracts were isolated from the supernatant after centrifugation at 15,000× *g* for 10 min. Equal amounts of protein extracts were separated by sodium dodecyl sulfate polyacrylamide gel electrophoresis (SDS-PAGE) and then transferred onto a polyvinylidene fluoride (PVDF) membrane (Millipore). The membrane was blocked with 5% skim milk at room temperature for 1 h, and then hybridized with a specific primary antibody with gentle agitation at 4 °C overnight. After washing with PBST (PBS containing 0.1% Tween-20), the membrane was incubated with HRP-conjugated secondary antibody at room temperature for 2 h. Subsequently, the tested proteins were visualized by the addition of enhanced chemiluminescence (ECL) re-agent (Millipore).

## 3. Results and Discussion

### 3.1. Mapping the Binding Interface between S100P and p53^1-73^ Region

The versatile NMR method uses chemical shift perturbation data to identify the interface region between protein-protein interactions [50]. In short, ^1^H-^15^N HSQC spectra are obtained for the labeled protein alone and then in combination with its target partner, which provides cross-peaks that have undergone chemical shift perturbation and/or a decrease in intensity. The affected cross-peaks provide information about the possible interface region and its associated residues. The interaction or protein binding to its target partner (other protein or ligand) influences the local environment of the nuclei present at or near the interface region thus showing the changes in cross-peaks located in the proximity of the interface region.

**Figure 1:**
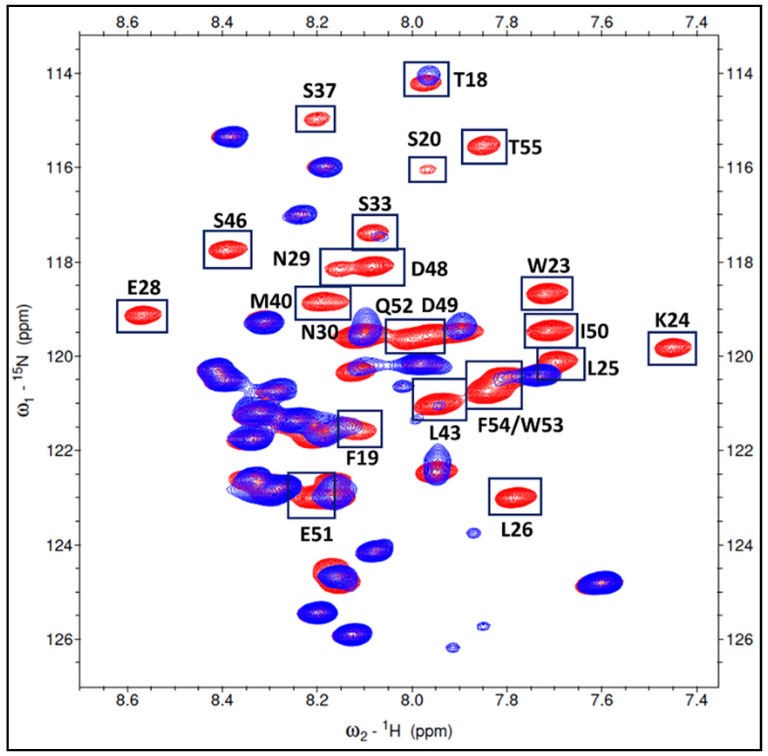
The overlaid of two HSQC spectra. The free ^15^N labeled p53^1-73^ spectrum was showed in red. The mix of the p53^1-73^ (with ^15^N labeled) and unlabeled S100P with 1:1 ratio was showed in blue. The cross peaks labeled with a box in this figure were the residues in p53^1-73^ domain that interacted with S100P.

The ^1^H-^15^N chemical shift assignments for the free S100P (BMRB 17866) and p53^1-73^ (BMRB 17760) proteins are available. We mapped the interacting p53^1-73^ region and the S100P residues near the interface region that formed the S100P-p53^1-73^ complex and were derived from the chemical shift perturbations and/or intensity decrease detected on the ^1^H-^15^N HSQC spectrum. At a 1:1 ratio of the ^15^N labeled p53^1-73^ to unlabeled S100P, a total of 26 residues corresponding to p53^1-73^ region (T18, F19, S20, W23, K24, L25, L26, E28, N29, N30, S33, S37, M40, D41, D42, L43, M44, S46, D48, D49, I50, E51, Q52, W53, F54, and T55) were identified and are shown in Figure 1.

**Figure 2:**
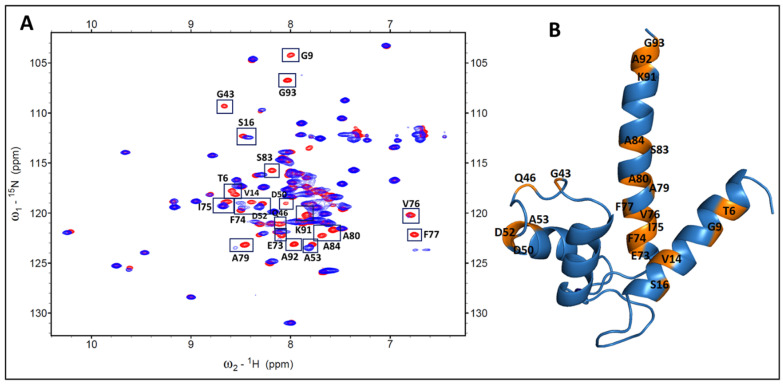
Analysis of the free S100P and S100P-p53^1-73^ complex using 2D NMR at a 1:1 binding ratio. (**A**) The overlaid 2D [^1^H-^15^N]-HSQC spectra highlight the spectral changes (showed with boxes) of the uniformly ^15^N-labeled S100P alone (shown in red) and ^15^N-labeled S100P upon binding to the unlabeled p53^1-73^ (shown in blue). (**B**) These boxed cross-peak residues (Figure 2A) were colored in orange and unbound residues were colored in blue on the cartoon structure of S100P using the PyMOL program.

Conversely, the addition of unlabeled p53^1-73^ to the ^15^N labeled S100P at a ratio of 1: 1 led to the identification of 21 residues (T6, G9, V14, S16, G43, Q46, D50, D52, A53, E73, F74, I75, V76, F77, A79, A80, S83, A84, K91, A92, and G93) in S100P that interacted with p53^1-73^ as shown in Figure 2A. These residues are also shown in orange color on the S100P cartoon structure prepared using the program PyMOL (Figure 2B).

### 3.2. Dissociation Constants Based on ^1^H-^15^N HSQC/NMR Titration Experiments

**Figure 3:**
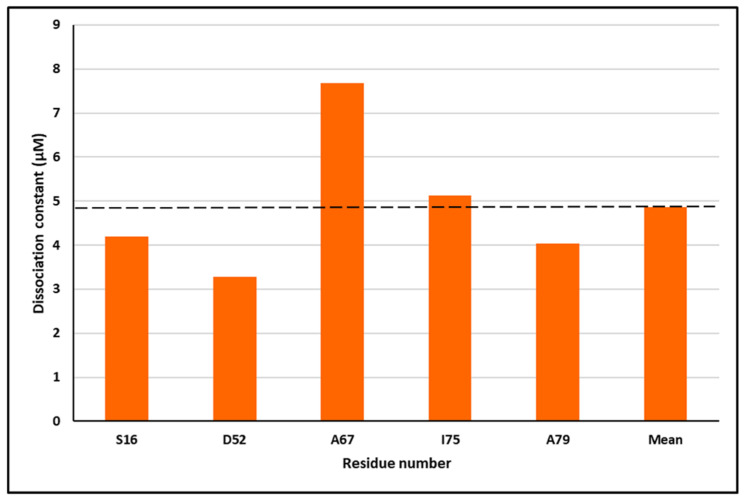
The dissociation constant (K_d_) measured using the specified residues found in ^15^N S100P titrations with unlabeled p53^1-73^, and the overall average K_d_ was 4.86 μM indicated with a broken line.

The data derived from the chemical shift perturbation analysis provided the values of the dissociation constants (K_d_). The cross-peaks under consideration were located in the fast exchange in both ^1^H and ^15^N dimensions. The ^15^N S100P HSQC/NMR spectra titrated with unlabeled p53^1-73^ indicated the cross-peaks that underwent significant alterations. The selected residues consisted of S16, M37, D52, A53, A67, I75, and A79. The mean K_d_ was found to be 4.86 μM as shown in Figure 3. These findings indicate that the resulting dissociation constant (K_d_) was in the micro-molar range.

### 3.3. Mapping the Binding Interface between S100P and Pentamidine

**Figure 4:**
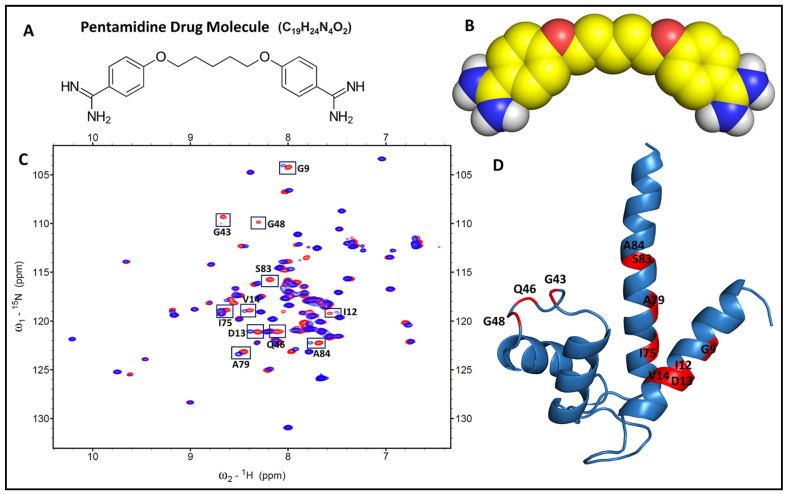
Analysis of the free S100P and S100P–pentamidine complex using 2D NMR at a 1:1 binding ratio. (**A**) The structural formula of pentamidine. (**B**) Sphere structure presentation of pentamidine. (**C**) The overlaid 2D [^1^H-^15^N]-HSQC spectra highlight the spectral changes (showed in boxes) of the uniformly ^15^N-labeled S100P alone (shown in red) and ^15^N-labeled S100P upon binding to unlabeled pentamidine molecules (shown in blue). (**D**) These selected cross-peak residues (boxed in Figure 4C) were colored in red and unbound residues were colored in blue on the cartoon structure of S100P using the PyMOL program.

A fingerprint of a protein’s conformational state is given by the ^1^H-^15^N HSQC spectrum. The chemical shift perturbation data indicative of cross-peaks alterations in ^1^H-^15^N HSQC spectra will easily distinguish the ligand-binding sites. Therefore, comparing the free and bound protein’s chemical shift disturbances (i.e., Δδ) is a useful tool for defining the protein–ligand binding interface. The overlapping of free ^15^N S100P and S100P in a complex with pentamidine spectra (Figure 4C) provides interesting information on the amino acid residues of S100P that interact with pentamidine. In principle, the residues showing the highest chemical shift perturbation reflects the most favored binding area for pentamidine. The S100P residues that appeared to participate in the interaction with pentamidine at a 1:1 ratio included G9, I12, D13, V14, G43, Q46, G48, I75, A79, S83, and A84. These residues are indicated in the HSQC spectrum of ^15^N S100P (Figure 4C) and are also highlighted with red color in the cartoon of S100P (Figure 4D). Figure 4A,B demonstrate the structural formula and spherical representation of the pentamidine drug molecule.

### 3.4. Dissociation Constants by NMR ^1^H-^15^N HSQC Titration Experiments

**Figure 5:**
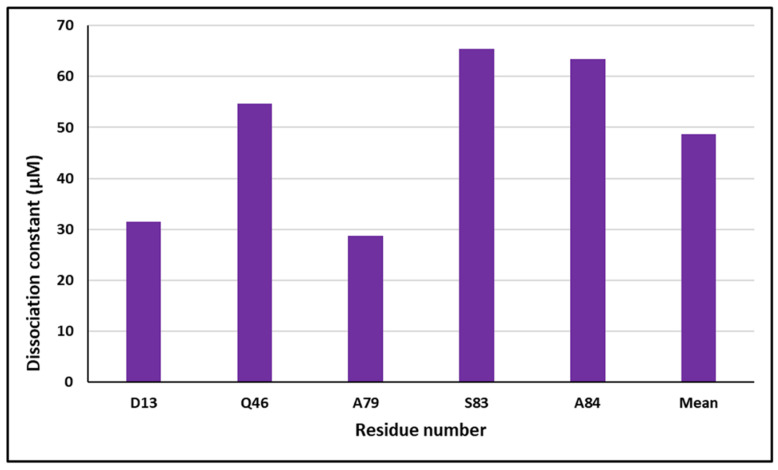
The dissociation constant (K_d_) measured using the specified residues found in pentamidine titrations with ^15^N S100P HSQC, and the overall average K_d_ was 48.74 μM as indicated with a broken line.

The data derived from the chemical shift perturbation analysis provided the values of the dissociation constants (K_d_). The cross-peaks under consideration were located in the fast exchange in both the ^1^H and ^15^N dimensions. The ^15^N S100P HSQC/NMR spectra titrated with unlabeled pentamidine indicated the cross-peaks undergoing significant alteration when compared with the free S100P protein. The selected residues consisted of D13, Q46, A79, S83, and A84. The mean K_d_ was found to be 48.74 μM as shown in Figure 5. These findings indicate that the resulting dissociation constant (K_d_) was in the micro-molar range.

### 3.5. Correlation of the Complex Structure Modules S100P-p53^1-73^ Region and S100P-Pentamidine

**Figure 6:**
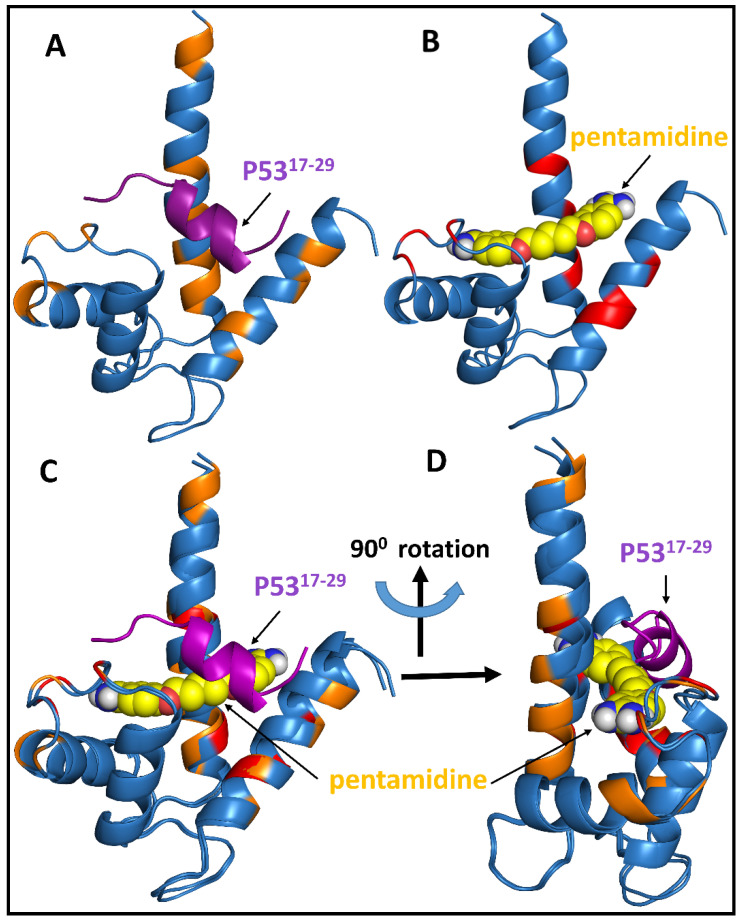
Two complex structures S100P-p53^17-29^ and S100P-pentamidine were superimposed. (**A**) The S100P-p53^17-29^ complex is shown in blue (S100P) and purple (p53^17-29^), and (**B**) the S100P-pentamidine complex is shown in blue (S100P) and yellow (pentamidine). (**C**) the overlapping structures of (**A**,**B**). (**D**) 90° angle rotation of (**C**). Figure 6D, pentamidine clearly blocked the binding between S100P (blue) and p53^17-29^ (purple).

The mapping of chemical shift data was, therefore, sufficient to identify the p53^1-73^ binding interface. For the S100P-p53^17-29^, we used the restrictions to measure the HADDOCK model. In the ^1^H-^15^N HSQC titration experiment, the resulting ambiguous interaction restraints (AIRs) were consistent with the major destructive chemical shifts of the residues. The binding area of p53^17-29^ was placed in the hydrographic cleft of S100P, and the S100P binding area included residues T6, G9, V14, S16, G43, Q46, D50, D52, A53, E73, F74, I75, V76, F77, A79, A80, S83, A84, K91, A92, and G93. The docking results show that the interaction between p53^17-29^ and S100P is regulated via hydrophobic interactions (Figure 6A).

The pentamidine interactions with S100P were characterized further by molecular docking, using ^1^H-^15^N HSQC spectra. By comparing the ^1^H-^15^N HSQC spectra of S100P in the absence and presence of pentamidine, we found that pentamidine-bound S100P residues in the S100P ^1^H-^15^N HSQC spectra from the perturbation or intensity decreased at cross-peaks as shown in Figure 4C. The mapping of these residues on a three-dimensional (3D) cartoon structure of S100P showed that these residues clustered in helix-1, a linker region, and helix-4 region (G9, I12, D13, V14, G43, Q46, G48, I75, A79, S83, and A84). Using the interaction sites as constraints, HADDOCK was again used to generate a potential model of the S100P–pentamidine complex (Figure 6B) using calcium-bound S100P (PDB code: 1J55) and pentamidine (Drug Bank, accession number DB00738). After the binding a complex of S100P-p53^17-29^ (Figure 6A) and S100P-pentamidine (Figure 6B) was obtained, and an overlapping model of these two binary complexes is shown in Figure 6C.

In the cell, p53 has biological function as tumor suppressor. It means that free p53 can prevent the cancer diseases. However, we found that free p53 could interact with S100P and form p53-S100P complex. When this complex is formed, p53 loses its biological function as a tumor suppressor. The small molecule of pentamidine could block the interaction between p53 and S100P. This means pentamidine could inhibit p53-S100P complex formation, and cancer disease can be prevented. Our docking findings indicate that pentamidine binds to an S100P region that strongly overlaps with the p53 binding site (Figure 6C).

### 3.6. The Effects of Pentamidine on Cell Proliferation and P53 Re-Activation in ZR-75-1 Breast Cancer Cells

The WST-1 cell proliferation assay provides an important strategy to check the efficacy of the proposed candidate molecule in in vitro environment. In this case, we propose that the pentamidine molecule would successfully disrupt the interactions between the S100P and p53 proteins, thereby influencing cell proliferation activity. Hence, to test our hypothesis we employed breast cancer derived ZR-75-1 cells since this cell line expresses high levels of functional endogenous p53 and S100P proteins. For better clarity, the gene expression of the S100P, S100B, and S100A4 were compared in glioblastoma and breast cancer cell lines by utilizing freely-available online database “Expression Atlas” facilitated by The European Bioinformatics Institute (EMBL-EBI). The comparison analysis indicated that the breast invasive ductal carcinoma cell line ZR-75-1 expresses the highest level of S100P and, therefore, the probability of pentamidine binding would favor S100P.

**Figure 7:**
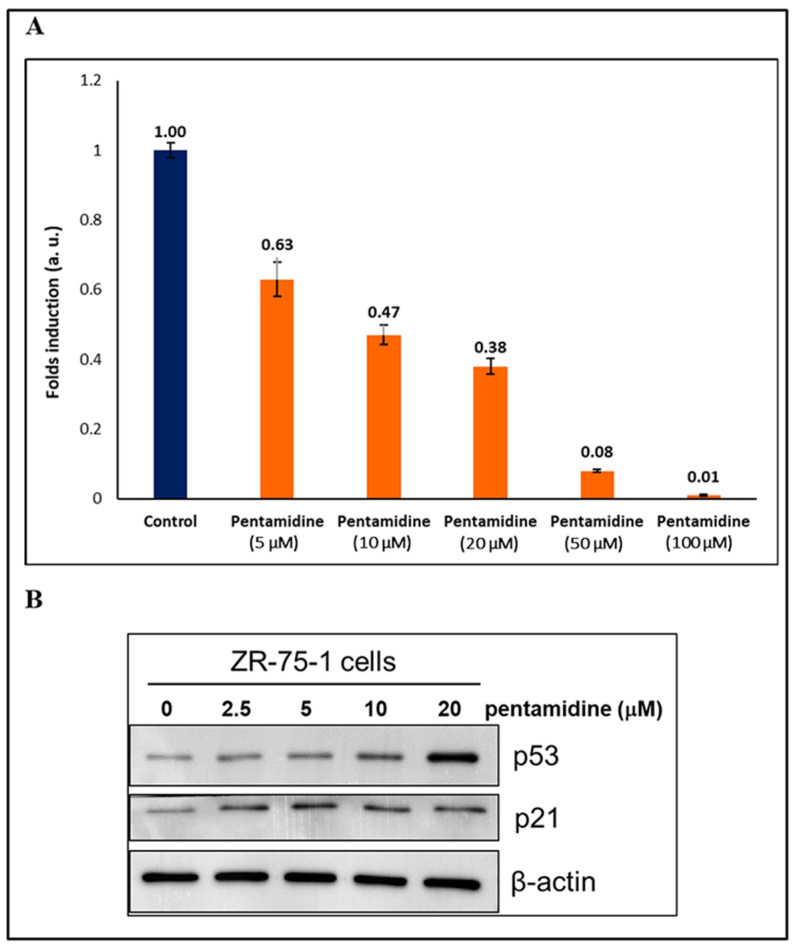
The effects of pentamidine on cell proliferation and p53 re-activation in ZR-75-1 breast cancer cells. ZR-75-1 cells were treated with indicated concentrations of pentamidine for 48 h. (**A**) Cell proliferation was determined by WST-1 assay. The bar diagram presented the relative folds of cell numbers in treated group (orange bars) comparing to that of untreated control group (blue bar). The values on the top of the bars were mean of three replicates. (**B**) Cell lysate was extracted from each treatment, and separated by SDS-PAGE, followed by Western blotting of p53, p21, and β-actin proteins. Beta-actin was a housekeeping protein used as the internal control of each treatment.

The ZR-75-1 breast cancer cells were treated with varying amounts of pentamidine (0, 5, 10, 20, 50, and 100 μM) for 48 h, and the relative folds of cell numbers were determined by the WST-1 assay. Figure 7A demonstrates the effect of pentamidine on the growth of cancer cells, and it can be observed that as the concentration of pentamidine increased, a corresponding cancer cell population decrease occurred. The observed effect was accompanied by a concentration-dependent behavior with increasing pentamidine concentrations leading to a substantial decrease in cell counts. Re-activation of the functional wild type p53 to induce p21, an inhibitor of cyclin-dependent kinases (CDKs), and thus suppress cell cycle progression, is a potential therapeutic strategy for cancers [51]. We also determined the protein levels of p53 and p21 after pentamidine treatment for 48 h. The results showed that pentamidine increased the amounts of p53 protein and p21 protein, a down-stream of p53, in a dose-dependent manner in ZR-75-1 human breast cancer cells (Figure 7B), suggesting that pentamidine might disrupt the interaction between p53 and S100P, leading to an increase and activation of functional wild type p53 and p21 pathway to reduce cell proliferation.

## 4. Discussion

Several posttranslational modifications, mainly through acetylation, methylation, and phosphorylation, determine the stability and function of the tumor suppression p53 protein. These alterations also establish the associations between p53 and its binding partners, which in turn affect modifications, stability, and behavior toward p53 [52]. The most significant changes were associated with the phosphorylation of three serine residues of p53 protein, which in the S100P-expressing cells was significantly reduced by 30% to 50%. Previous research papers clearly indicate that the S100P mediated the inactivation of p53 because the phosphorylated N-terminal Ser15 and Ser 46, in particular, strongly influenced the potential for transactivation of p53 [53,54]. In this study, we include the combination of structural biology, molecular biology, and computational chemistry approaches for characterizing the interactions between S100P-p53TAD and S100P-pentamidine. The interactions are described by the use of techniques, such as NMR spectroscopy and HADDOCK. ^1^H-^15^N HSQC/NMR spectroscopy was chosen as it provides the mapping of the protein-protein and the protein-ligand binding interface directly to the surface of the protein structure.

In the present study, we first concentrated on the structural determinants of specific protein-binding interfaces; thus, for that purpose, we performed NMR ^1^H-^15^N HSQC titration, and HADDOCK to evaluate the binding of S100P with the p53^1-73^ and pentamidine. The S100P binding region of the p53^1-73^ overlaps with the binding sites of the pentamidine molecules, which shows that pentamidine could block the interaction of the S100P and p53^1-73^ region. In the second part the concept was proved in ZR-75-1 human breast cancer cells. We have shown that blocking the interaction of S100P and p53 by pentamidine results in the stabilization of p53 at the protein level and induction of its down-stream gene, p21, thereby inhibiting cell proliferation (Figure 7). Capoccia, E. et al. have reported that pentamidine inhibits S100B-p53 interaction in rat C6 glioma cells, which results in significant decreases in proliferation, migration, and apoptosis [55]. Recently, we demonstrated that pentamidine effectively disrupts the interaction of S100A4 and p53 and inhibits cell proliferation in MCF-7 human breast cancer cells [56]. Thus, we cannot exclude the possibility that pentamidine targets to other p53-associated proteins, such as S100A4 and S100B, and disrupts their interactions in ZR-75-1 cells. Based on the mRNA expression from RNA-sequencing database (Expression Atlas- as mentioned earlier), S100P (212 TPM: Transcripts Per Million) might be the dominant target of pentamidine rather than S100A4 (18 TPM) and S100B (0.6 TPM) in ZR-75-1 cells. This research will concentrate on the potential roles of S100P and p53 in cancer. We will also discuss the possible advantages of developing new drug targets to inhibits S100P’s role in cancer tissue.

In conclusion, we demonstrated that pentamidine is an effective agent that can be used to inhibit cancer progression. The findings show that pentamidine could help to treat cancer as an anti-cancer agent or as a targeted therapy along with some other approaches. The pentamidine molecule constitutes the first step of a logical drug design project aimed at identifying the S100P protein inhibitor that would bind to S100P at the p53 binding site. In future work, these lead compounds will be developed into higher-affinity S100P inhibitors that could possibly have useful therapeutic properties.

## Data Availability

Not applicable.

## Supplementary Materials

The following are available online at https://www.mdpi.com/article/10.3390/biom11050634/s1, Figure S1: Overlays of the ^15^N-^1^H HSQC spectra of free 0.3 mM [^15^N] S100P in complex with various concentrations of p53^1-73^ region in the presence of 1:0.25 (gold), 1:0.5 (blue), 1:1 (green), and 1:2 (cyan) are shown. Figure S2: Overlays of the ^15^N-^1^H HSQC spectra of free 0.3 mM [^15^N] S100P in complex with various concentrations of pentamidine in the presence of 1:0.25 (blue), 1:0.5 (green), 1:1 (gold), and 1:2 (cyan) are shown. Figure S3: Overlays of the ^15^N-^1^H HSQC spectra of free 0.3 mM [^15^N] p53^1-73^ in complex with various concentrations of S100P proteins. in the presence of 1:0.25 (blue), 1:0.5 (gold), 1:0.75 (green), 1:1 (cyan), and 1:2 (brown) are shown.
