# Supplementary material for: S100P Interacts with p53 while Pentamidine Inhibits This Interaction
Source: Biomolecules. 2021 Apr 24;11(5):634. doi: 10.3390/biom11050634 (PMC8145327; doi:10.3390/biom11050634)
Supplement: Supplementary file 1 [file biomolecules-11-00634-s001.zip › biomolecules-1170271-supplementary.pdf]

Supplementary Materials:

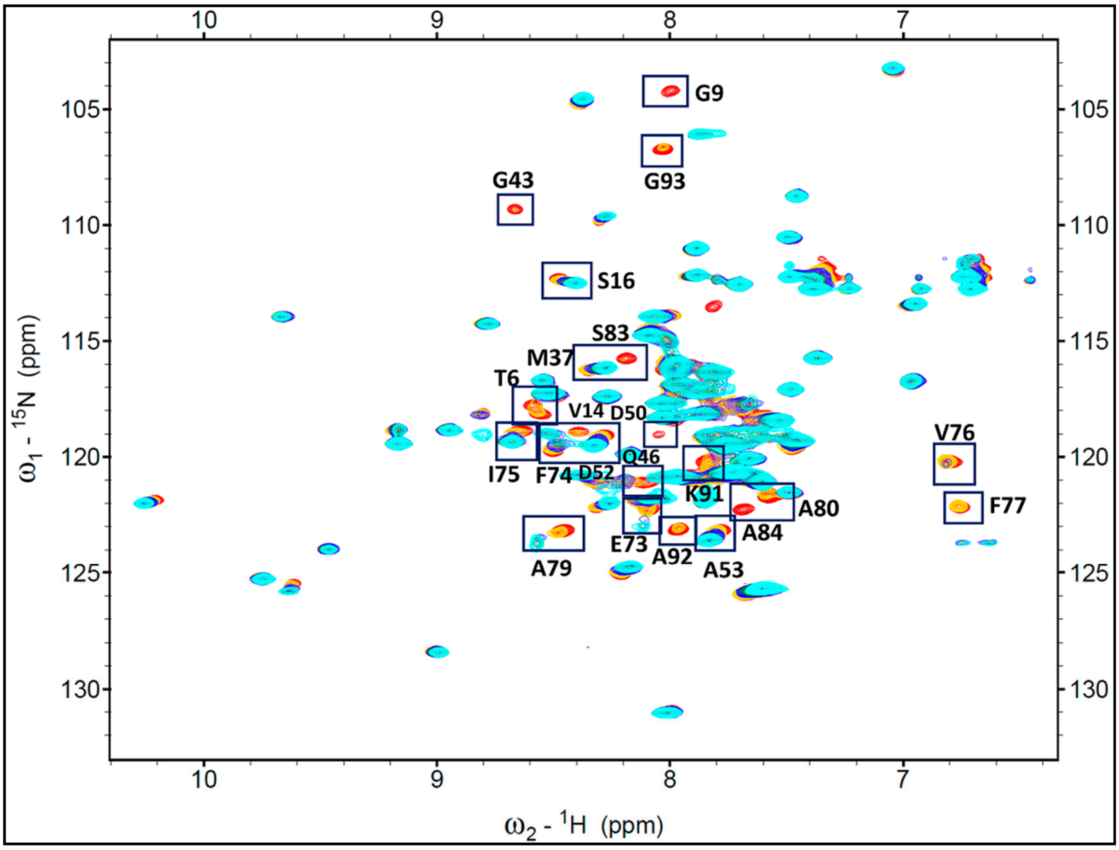

Figure S1. Overlays of the ^{15}N-^{1}H HSQC spectra of free 0.3 mM [^{15}N] S100P in complex with various concentrations of $p53^{1–73}$ region proteins in the presence of 1:0.25 (gold), 1:0.5 (blue), 1:1 (green), and 1:2 (cyan) are shown.

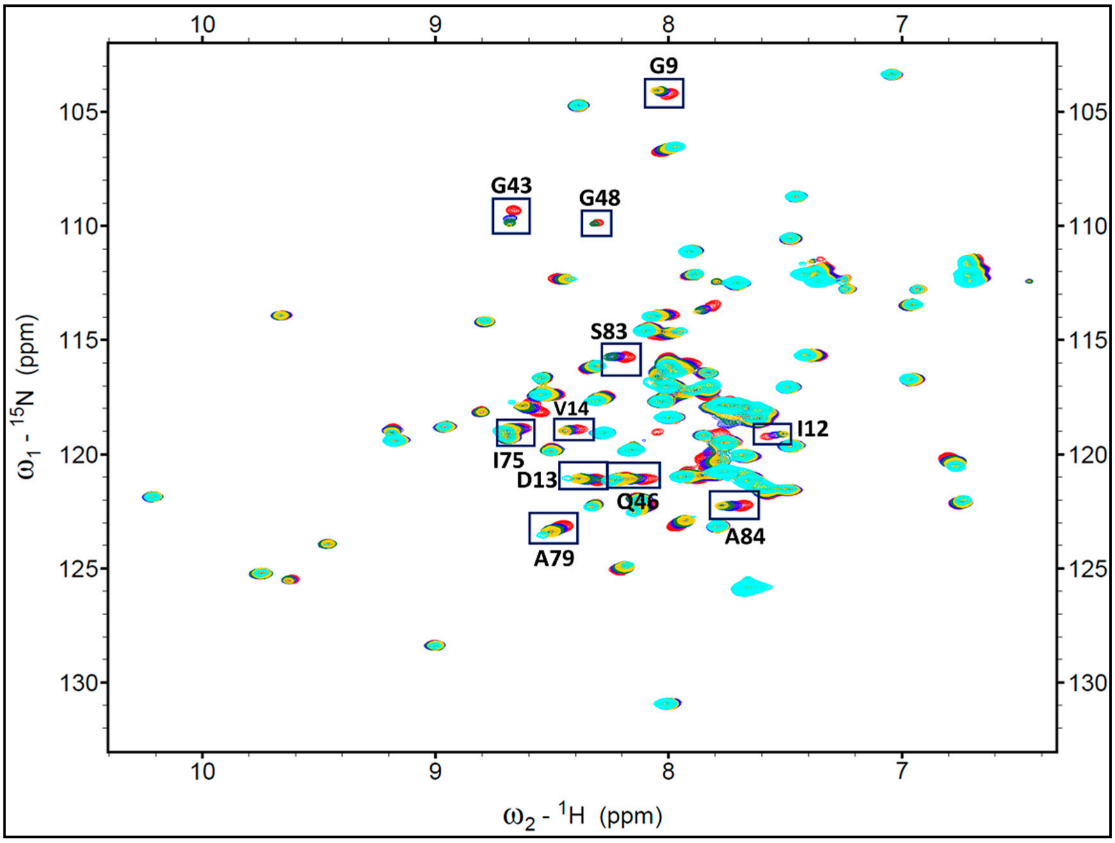

Figure S2. Overlays of the ^{15}N-^{1}H HSQC spectra of free 0.3 mM [^{15}N] S100P in complex with various concentrations of pentamidine in the presence of 1:0.25 (blue), 1:0.5 (green), 1:1 (gold), and 1:2 (cyan) are shown.

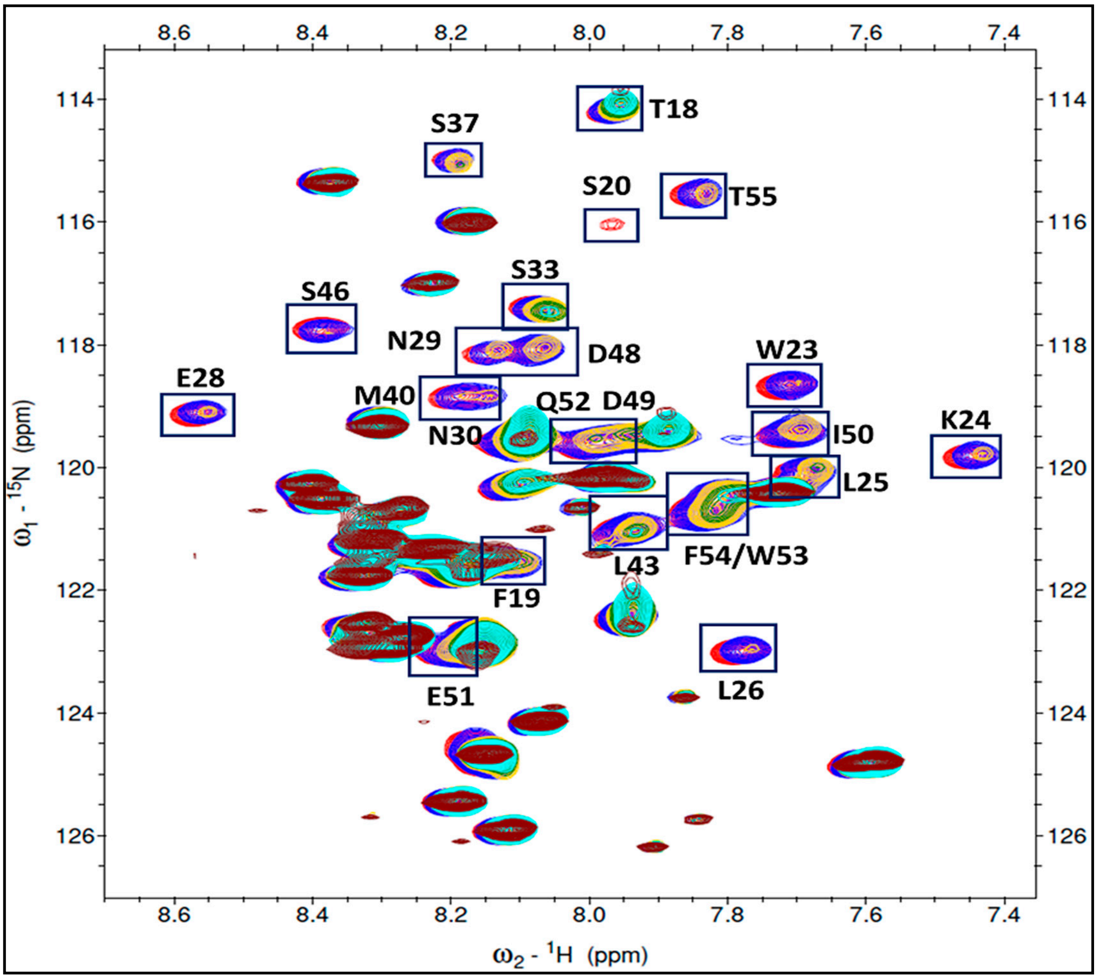

Figure S3. Overlays of the ^{15}N-^{1}H HSQC spectra of free 0.3 mM [^{15}N] $p53^{1-73}$ in complex with various concentrations of S100P proteins in the presence of 1:0.25 (blue), 1:0.5 (gold), 1:0.75 (green), 1:1 (cyan), and 1:2 (brown) are shown.
